# The impact of exercising self-efficacy on physical education learning engagement: the mediating role of flow experience

**DOI:** 10.3389/fpsyg.2025.1588643

**Published:** 2025-06-04

**Authors:** Wenzhe Huang, Ruikai Wei, Bojin Cheng

**Affiliations:** ^1^School of Arts and Sports, Shantou Preschool Education College in Guangdong, Shantou, China; ^2^Student Affairs Office, Shantou Preschool Education College in Guangdong, Shantou, China; ^3^School of Physical Education, Guangzhou Sport University, Guangzhou, China

**Keywords:** physical education learning engagement, exercising self-efficacy, flow experience, structural equation model, college student

## Abstract

**Introduction:**

The study aims to examine the direct and indirect effects of exercising self-efficacy on physical education learning engagement through mediator flow experience.

**Methods:**

Based on a questionnaire survey, using the Exercise Self-efficacy Scale, Flow Experience Scale, and Physical Education Learning Engagement Scale, a survey was conducted among 568 university students from five universities in Guangdong Province, China.

**Results:**

① There were significant positive correlations between exercise self-efficacy, flow experience, and physical education learning engagement; ② Exercise self-efficacy positively influenced physical education learning engagement; ③ Flow experience played a mediating role between exercise self-efficacy and physical education learning engagement.

**Discussion:**

This study fills the theoretical gap of flow experience application in the context of physical education, and uses the variable of exercise self-efficacy, which combines individual self-efficacy with the field of physical exercise, to enhance the depth and strength of explanation and prediction. The findings of this study contribute to physical education instructors’ understanding of the psychological mechanisms at play in college students’ physical education learning process. Additionally, the examination of the pivotal mediating role of flow experience offers fresh perspectives for enhancing the efficacy of physical education instruction.

## Introduction

The COVID-19 pandemic has precipitated a notable decline in university students’ physical fitness and health status, which has emerged as a pressing issue requiring immediate scholarly attention and targeted intervention strategies. For example, in China, the Release of the Eighth National Student Physical Fitness and Health Survey Results (Chinese School Health, 2021) reveals differential improvements in the excellent-good attainment rate of physical health standards across age groups: a 5.1-percentage-point increase among adolescents (13–15 years), 1.8 percentage points for senior high school students (16–18 years), and merely 0.2 percentage points for university cohorts (19–22 years), indicating the least significant progress in the collegiate demographic. Schools physical education are uniquely positioned to address the epidemic of physical inactivity among college student, which is also the main way to guide college students to form health awareness and scientific fitness concepts. Physical education learning engagement is an important indicator for measuring students’ participation in the implementation of physical education courses. It refers to individuals actively striving to master knowledge and skills, and is specifically manifested in their preferences for sports activities and active participation in the process of physical education learning (Bevans et al., 2010). In recent years, the investment in sports learning and its influencing factors have received increasing attention, with self-efficacy being a major concern. The theory of learning engagement states that individual self-efficacy is an important variable for improving effort levels (Skinner et al., 2009). Research has found that self-efficacy, as a belief in learners’ abilities, not only affects students’ emotional states during physical education learning, but also influences the attribution of physical education learning outcomes. It plays a key role in regulating individuals’ efforts and persistence in physical education learning behavior and is an important proximal factor affecting learning engagement (Jiang et al., 2019). Although academic research has focused on self-efficacy and learning engagement, there are still some shortcomings. For example, in terms of research tools, some studies have examined the impact of general self-efficacy on sports learning engagement (Yang et al., 2024). However, Bandura, the proponent of self-efficacy theory, emphasizes that contextualized measurements often have limited explanatory and predictive value, and accurate judgments of self-efficacy should be based on specific domains or task (Pajares, 1996; Everett et al., 2009). Therefore, for physical education learning engagement, exercise self-efficacy, which refers to an individual’s belief or confidence in overcoming internal and external obstacles and persisting in exercise behavior (Allison et al., 1999; Roesch et al., 2013). This variable that combines individual self-efficacy with the field of physical exercise provides better depth and intensity of explanation and prediction. Therefore, this study proposes the hypothesis that:

**Hypothesis 1**: Exercise self-efficacy positively affects physical education learning engagement.

With the rise of positive psychology, exploring college students’ learning engagement from the perspective of flow experience has received widespread attention and research. Flow experience refers to the optimal emotional experience or state in which an individual exhibits a strong interest in an activity and is fully engaged in solving a problem or participating in an activity (Csikszentmihalyi, 2000). It originated from observing physical activities such as rock climbing and dancing (Jiang et al., 2021). However, in recent years, research on the impact of flow phenomenon on learning engagement has mostly focused on online activities such as remote live streaming, virtual reality teaching, and online learning (Ran, 2017; Cai and Jia, 2020). However, there is little research on whether and how it promotes learning engagement in the field of physical education teaching. The flow theory states that the key prerequisite for entering flow is to establish a balance between an individual’s perceived ability to act and the opportunity to act (Chen, 2014), while self-efficacy is the individual’s expectation and judgment of their ability to participate in activities and complete goals and tasks. Therefore, related research has also proposed that self-efficacy is an important prerequisite for the experience of flow (Srivastava et al., 2010). In addition, flow theory suggests that flow can affect an individual’s level of engagement and behavioral intention towards the activity, as the self motivation mechanism of flow causes individuals to involuntarily invest more time and energy (Chen, 2014). A cross-border study found that the more confident students are in their learning abilities, the more likely they are to experience flow during the learning process, and their learning engagement will also increase accordingly (Mesurado et al., 2016). A study on desktop virtual reality teaching environment in China conducted multimodal measurement and analysis of students’ physiological data, and found that self-efficacy positively affects flow experience, which in turn has a positive impact on learning outcomes including cognition and emotion (Liu et al., 2022). It can be inferred that students’ exercise self-efficacy will affect the generation of central flow experience during physical education classes, thereby affecting their engagement in physical education learning. Based on this, this study proposes the following hypotheses:

**Hypothesis 2**: Exercise self-efficacy positively affects flow experience.

**Hypothesis 3**: Flow experience positively affects physical education learning engagement.

**Hypothesis 4**: Exercise self-efficacy will predict the physical education learning engagement through the mediating role of flow experience.

## Method

### Participants

The sampling method in this study is to classify universities in Guangdong Province, China according to education level and type, and then perform convenience sampling. The final sample comprised non-physical-education majors recruited from five universities (South China Normal University, Zhaoqing College, Xinghai Conservatory of Music, Guangdong Industry Polytechnic University, Guangdong Finance and Trade Vocational College). As physical education courses in Chinese universities are public elective courses for freshmen and sophomores, this study only targets freshmen and sophomores. Physical education courses involve sports such as table tennis, badminton, basketball, football, and aerobics, which have good representativeness. To guarantee the robustness of the sample estimation, the minimum sample size should be 10 times the number of scale measurement items in the model (Haenlein and Kaplan, 2004). Given that the number of items in this study is 29, the minimum required sample size is 290. A total of 656 questionnaires were distributed, and after excluding questionnaires with short answer times and regular responses, 568 valid questionnaires were finally collected, with a valid questionnaire collection rate of 86.59%, which significantly exceeding the minimum sample size. The demographic variables such as gender and grade are detailed in Table 1.

**Table 1:** Description of demographic variables.

Variable	category	Frequency	percentage (%)
Gender	Male	261	46.0
	Female	307	54.0
Grade	Freshman	251	55.8
	Sophomore	317	44.2
Education	Undergraduate	373	65.7
	Junior college	195	34.3
University Type	Science and engineering	129	22.7
	Normal	52	9.2
	Professional	146	25.7
	Comprehensive	241	42.4

### Procedure

After obtaining informed consent from physical education teachers and students themselves, group testing will be conducted on a class by class basis during physical education classes. To further enhance the rigor of the research, standardized guidelines and anonymous responses were used, and questionnaires were filled out and collected on the spot. During the investigation, the principles of voluntary participation, data confidentiality, and anonymity were emphasized, and we controlled for demographic variables including the gender and grade of participants.

### Measures

#### Exercise self-efficacy scale

We adopted the Chinese version of the Exercise Self-Efficacy Scale, compiled by Motl et al. (2000). and revised by Chen et al. (2019). This scale is unidimensional and consists of 8 items, using a 5-point Likert scale ranging from “1” (strongly disagree) to “5” (strongly agree), with intermediate scores of 2, 3, and 4. The higher the total score from all items, the stronger the student’s exercise self-efficacy.

#### Flow experience scale

We adopted the Flow Experience Scale developed by Chang and Zhu (2012). This scale is unidimensional and consists of 4 items, using a 5-point Likert scale ranging from “1” (strongly disagree) to “5” (strongly agree), with intermediate scores of 2, 3, and 4. The higher the total score from all items, the stronger the student’s flow experience.

#### Physical education learning engagement scale

We adopted the Physical Education Learning Engagement developed by Fang et al. (2008) and revised by Cheng et al. (2022). This scale includes 17 questions in three dimensions: vigor, dedication, and absorption, using a 5-point Likert scale ranging from “1” (strongly disagree) to “5” (strongly agree), with intermediate scores of 2, 3, and 4. The higher the total score from all items, the stronger the student’s physical education learning engagement.

### Data analysis

Descriptive statistical analysis and correlation analysis of the collected data were conducted using SPSS 26.0 software; common method bias testing, confirmatory factor analysis (CFA). Examination of the effect values of the mediation model constructed for this study were performed using Amos 24.0 software, and alidate the constructed mediation model using Bootstrap method.

## Results

### Common method bias test

Due to the fact that this study only employs a questionnaire survey method, there may be a certain degree of Common Method Bias (CMB). Therefore, this research employs both procedural control and statistical testing methods to minimize the negative impact of CMB on the accuracy of the research findings and to ensure the reliability of the model data analysis. In terms of procedural control, following the suggestions of scholar Podsakoff et al. (2012), firstly, it is clarified that there are no right or wrong answers to each question, and there are no ambiguous or unclear expressions. The order of the questions has been reasonably arranged, the measurement significance of the items has been concealed, and the duration of questionnaire completion has been controlled. Secondly, participants were informed in advance that the survey data would only be used for academic research, and that all responses would be anonymous. They were also emphasized that their instructors would not be aware of their answers, encouraging them to provide truthful responses based on their own situations, thereby reducing social desirability bias and effectively minimizing data distortion.

In terms of statistical testing methods, following the suggestions of Tang and Wen (2020), the first step is that this study employs the CFA comparison method to further test for CMB. Firstly, Model 1 is constructed as a single-factor structure consisting of all items, while Model 2 represents the theoretically complete correlation structure of the CFA in this study. The testing is conducted by comparing the difference in degrees of freedom and chi-square values between Model 1 and Model 2. Initially, the fit indices for Model 1 are not ideal, with χ2/df = 28.940, GFI = 0.488, NFI = 0.677, IFI = 0.685, TLI = 0.631, CFI = 0.684, RMSEA = 0.222. Secondly, as shown in Table 2, under the condition of 6 degrees of freedom and a 95% confidence interval, the calculated lower limit of the △χ2 for the two models is 7.81, while the △χ2 in this study is 2146.88, which far exceeds the critical value, indicating to some extent that the CMB present in this study is not significant.

**Table 2:** Summary table of chi-square values and degrees of freedom changes in CFA comparison method.

Model	*χ^2^*	*df*	△*χ^2^*	△*df*	*p*
Single factor	2604.61	90	2146.88	3	0.05
Multi-factor	457.73	87			

### Reliability and validity testing and confirmatory factor analysis

Given satisfactory model fit indices (CFI = 0.953, TLI = 0.944, IFI = 0.954, NFI = 0.943, GFI = 0.902, RMSEA = 0.087), the analysis proceeded as follows: First, internal consistency was assessed using Cronbach’s α coefficients. All dimensions demonstrated α values ranging from 0.861 to 0.956 (Table 3), exceeding the threshold of 0.7, thereby confirming the questionnaire’s robust reliability. Second, construct validity was evaluated through composite reliability (CR) and average variance extracted (AVE). As shown in Table 3, all CR values (0.905–0.971) surpassed the 0.7 benchmark, while AVE values (0.708–0.869) exceeded the 0.5 criterion. These results collectively indicate adequate convergent validity and composite reliability across measurement items.

**Table 3:** Results of reliability and validity tests.

Variable	Item	Factor Loading	CR	AVE	Cronbach’s α
Exercise Self-efficacy	es1	0.923	0.964	0.757	0.941
	es2	0.733			
	es3	0.946			
	es4	0.918			
	es5	0.793			
	es6	0.836			
	es7	0.862			
	es8	0.928			
Flow Experience	fe1	0.886	0.905	0.708	0.861
	fe2	0.886			
	fe3	0.649			
	fe4	0.916			
Vigor	vi1	0.836	0.959	0.795	0.937
	vi2	0.914			
	vi3	0.902			
	vi4	0.827			
	vi5	0.911			
	vi6	0.952			
Dedication	de1	0.918	0.971	0.869	0.956
	de2	0.945			
	de3	0.954			
	de4	0.912			
	de5	0.931			
Absorption	ab1	0.891	0.968	0.836	0.949
	ab2	0.913			
	ab3	0.894			
	ab4	0.921			
	ab5	0.944			
	ab6	0.923			

### Descriptive statistics and correlation analysis

This study used Pearson correlation analysis. As shown in Table 4, there is a significant positive correlation between Exercise self-efficacy, flow experience, vigor, dedication, and absorption. These relationships between variables support the subsequent testing of our hypotheses.

**Table 4:** Means, standard deviations, and correlations among study variables.

Variable	*M*	*SD*	1	2	3	4	5
Exercise self-efficacy	3.33	0.88	1.00				
Flow experience	3.77	0.78	0.54**	1.00			
Vigor	3.51	0.84	0.67**	0.72**	1.00		
Dedication	3.78	0.80	0.61**	0.71**	0.87**	1.00	
Absorption	3.67	0.80	0.61**	0.73**	0.86**	0.90**	1.00

**p < 0.01.

Since all the scales employed in this study are well-established, the analysis results indicate a high degree of reliability. To achieve a more streamlined model and enhance the stability of parameter estimation, this study utilized item parceling for the multidimensional variable (physical education learning engagement) in the initial model, which involves combining two or more items from the scale into a new indicator, with the mean score serving as the basis for analysis of this new indicator (Wu and Wen, 2011).

According to the mediation effect testing process suggested by Wen et al. (2018), we examined the mediating role of flow experience between exercise self-efficacy and physical education learning engagement. First, we tested the direct path of exercise self-efficacy on physical education learning engagement. Before including the mediator variable, exercise self-efficacy significantly positively influenced physical education learning engagement (β = 0.53, *p* < 0.001), thus Hypothesis 1 was supported. After including the mediator variable, all path standardized coefficients remained significant (*p* < 0.05). Exercise self-efficacy positively predicted physical education learning engagement (β = 0.18, *p* < 0.001), exercise self-efficacy positively predicted flow experience (β = 0.68, *p* < 0.001), and flow experience positively predicted physical education learning engagement (β = 0.35, *p* < 0.001), thus Hypotheses 2 and 3 were supported.

**Figure 1:**
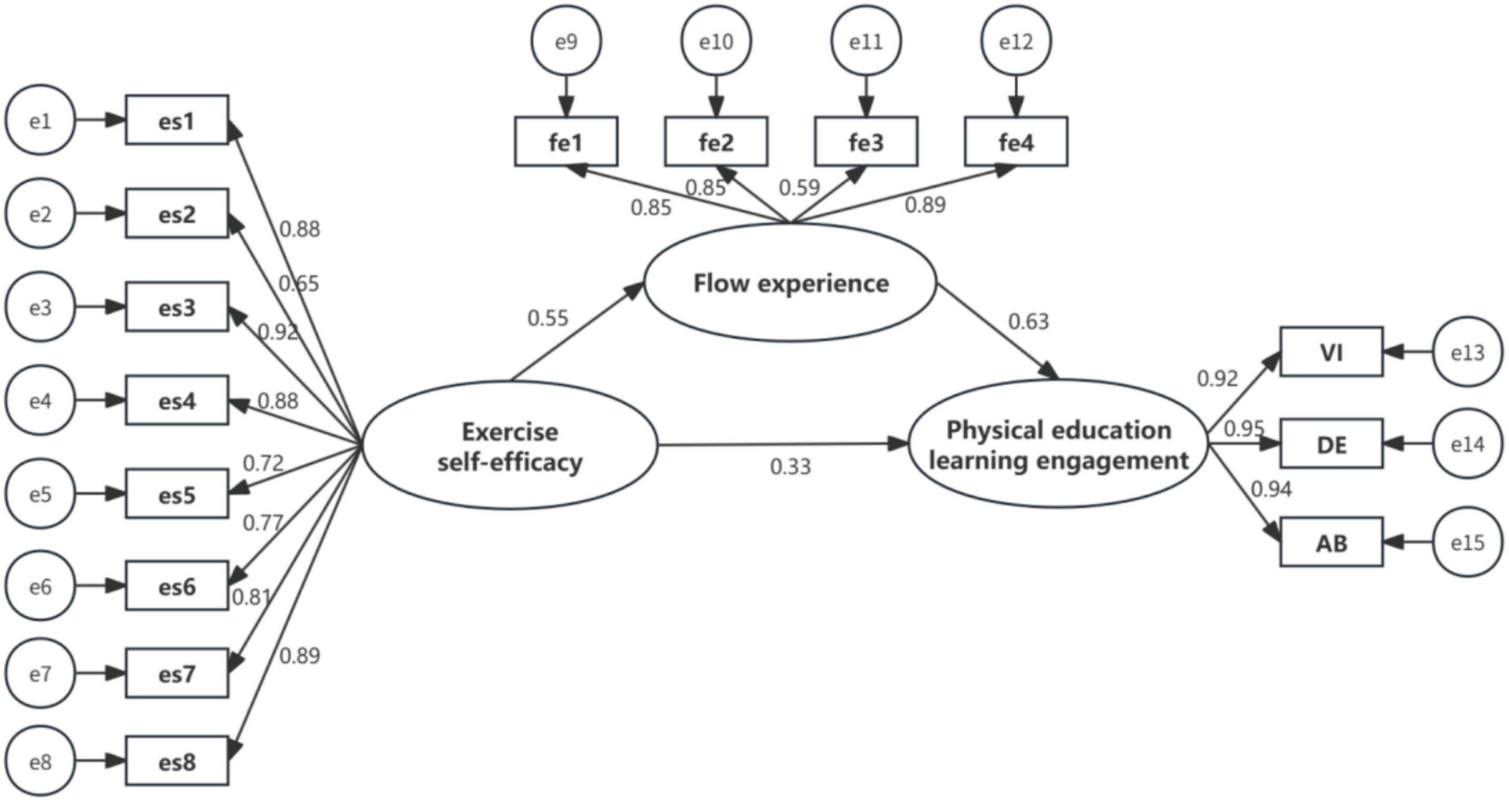
The mediating effect of flow experience in the relationship between exercise self-efficacy and physical education learning engagement.

The subsequent step involves investigating the mediating role of flow experience in the relationship between exercise self-efficacy and physical education learning engagement. To achieve this, a resampling frequency of 5,000 iterations was employed to conduct bootstrapping analysis on the original dataset. Both the Bias-corrected method and the Percentile method were utilized for hypothesis testing. If zero was not included within the 95% confidence interval and the *p*-value was less than 0.01, it would indicate that the indirect effects of the model were significant, thereby confirming the mediating effect of flow experience. As shown in Table 5: The size of the indirect effect of flow experience is 0.28. The 95% confidence intervals obtained from the Bias-corrected method and the Percentile method are [0.23, 0.33] and [0.23, 0.34], respectively, with *p* < 0.001. These results confirm the significant mediating effect of flow experience, supporting Hypothesis 4. Furthermore, given that the direct effect of exercise self-efficacy on physical education learning engagement remains significant, flow experience is determined to play a partial mediating role (Figure 1).

**Table 5:** Mediation effect and effect size.

Path	Effect	SE	Bias-corrected 95%CI			Perentile 95%CI		
			Lower	Upper	*p*-value	Lower	Upper	*p*-value
ES → PELE	0.26	0.03	0.20	0.33	***	0.20	0.33	***
ES → FE	0.36	0.03	0.28	0.42	**	0.29	0.42	***
FE → PELE	0.78	0.06	0.67	0.93	**	0.67	0.94	***
ES → FE → PELE	0.28	0.03	0.23	0.33	**	0.23	0.34	***

ES, exercise self-efficacy; FE, flow experience; PELE, physical education learning engagement. **p < 0.01; ***p < 0.001.

## Discussion

### The direct effect of exercising self-efficacy on physical education learning engagement

The results of this study show that college students’ exercising self-efficacy significantly and positively affects physical education learning engagement, consistent with existing research (Cheng et al., 2022; Li, 2022). However, with regard to research tools, existing studies have predominantly examined the influence of general self-efficacy on physical education learning engagement. While it demonstrates a certain level of predictive capability, this study integrates self-efficacy within the domain of physical exercise and further investigates its relationship with physical education learning engagement. This approach will enhance both the depth of explanation and the precision of prediction.

Bandura’s self-efficacy theory suggests that an individual’s subjective perception of their own abilities influences the effort and persistence of their corresponding behavior (Bandura, 1977). Self-efficacy regulates behavior performance by affecting cognition, emotion, and choice (Deci and Ryan, 2013). In the context of physical education, high self-efficacy in exercise helps students actively engage with the sports techniques taught by their instructors, set higher athletic goals, view difficulties encountered during practice as challenges, and respond positively, exhibiting greater enthusiasm and persistence in learning. Conversely, students with low exercise self-efficacy tend to adopt a self-handicapping mindset towards learning sports techniques, lacking high engagement and effort in physical education tasks. Faced with adverse situations in the learning process, they often choose to evade, showing insufficient learning engagement. Our survey also found that individuals with high exercise self-efficacy perform better in sports and have a certain pursuit of physical exercise, both of which promote engagement in physical education. This suggests that emphasizing the development of students’ sense of physical exercise competence helps to enhance their involvement in physical education and maintain a high level of commitment to physical education learning.

### The mediating effect of flow experience

The results of this study indicate that flow experience, as an interactive reaction between students and physical education classes, plays an important mediating role in the mechanism by which exercising self-efficacy affects physical education learning engagement, aligning with previous research findings (Liu et al., 2022; Mesurado et al., 2016). However, most existing research on the role of flow experience in education and teaching has primarily concentrated on learning environments dominated by knowledge acquisition, overlooking those environments where dominated by physical activity and skill learning. Consequently, this study delves into the significant role of flow experience in the context of physical education instruction, thereby enhancing the flow theory.

Some scholars believe that flow has a self-motivating mechanism that makes individuals in a flow state highly focused on the activities they participate in (Chen, 2014). This study also found that students who enter a flow state are more likely to become deeply engaged in physical tasks, exhibiting higher learning engagement. Additionally, flow theory posits that individuals are more likely to be fully immersed and experience a sense of transcendence when there is a balance between perceived ability and task difficulty. This specific reaction significantly regulates an individual’s emotional state, thereby influencing their participation decisions, a viewpoint that was also verified during qualitative interviews in this study. Thus, exercising self-efficacy is an important antecedent variable for college students entering a flow state during physical education, and whether they enter this state affects their emotional engagement in learning sports techniques. However, previous research related to physical education has rarely addressed flow experience, overlooking students’ cognitive feedback on the smoothness of the learning process, goal completion, and activity control, thereby greatly diminishing the effectiveness of physical education and hindering the cultivation of students’ interest in sports. Therefore, examining the critical mediating role of flow experience provides new insights for improving the effectiveness of physical education.

It is worth noting that the prerequisite for generating flow experience is the balance between personal skills and challenges. If the challenge exceeds the skill, the individual becomes anxious; if the skill surpasses the challenge, the individual initially relaxes and soon feels bored and weary. Due to the unique nature of physical education and the varying physical fitness levels of students, the relationship between skills and challenges varies greatly among individuals. For example, a simple physical task might seem extremely challenging to students who lack regular exercise, while it might be too easy for those adept at sports, failing to effectively stimulate their neural networks or elicit psychological responses. Therefore, how physical education content matches students’ perceived skills, and how to balance skills and challenges, is crucial for triggering flow and enhancing engagement in physical education classes.

### Research recommendations and limitations

In summary, this study applies flow theory to university physical education classes, examining the internal mechanisms by which exercise self-efficacy influences physical education learning engagement, and has certain practical guiding significance for physical education teaching practices in universities. Firstly, fostering college students’ sense of physical exercise competence and bolstering their confidence in acquiring sports skills serve as prerequisites for enhancing physical education learning engagement. Physical education instructors should prioritize hierarchical and flexible teaching methods, progressing from simple to complex tasks. They should also encourage students to experiment with techniques, provide timely and positive feedback, guide them toward gradual success, and accumulate targeted successful experiences. The accumulation of multiple successful experiences can foster a high level of self-confidence in learning sports skills. Secondly, optimizing classroom interaction between teachers and students and balancing abilities with challenges is crucial. Given the pivotal role of flow experience in promoting engagement in physical education learning, physical education instructors should fully leverage the positive outcomes of flow experience, such as intense concentration, enjoyment, and a sense of control, to enhance students’ engagement. Specifically, it is essential to improve classroom management skills while prioritizing students’ emotional experiences. The arrangement of physical education content should emphasize fun and interactivity, guiding students to enter a state of flow, immerse themselves in learning, and achieve mastery through immersion. Moreover, striking a balance between the challenge level of motor skills and students’ skill proficiency is key to achieving a flow experience. It is imperative to create appropriate task-mastery atmospheres tailored to individual differences in students’ physical conditions and athletic foundations.

However, this study also has the following limitations. Firstly, the questionnaire survey used in the study is a cross-sectional research method, making it difficult to verify the causal relationships between variables. Future research can further investigate through experimental research or longitudinal tracking studies. Secondly, the variables in the study are all self-reported by students. Future research can enrich data collection methods and use a combination of questionnaire scales and physiological data for subjective and objective measurement analysis to improve the stability of research results. Finally, the mechanisms of exercise efficacy and learning engagement may differ between male and female students, and the degree of flow experience may vary across different sports. Future research will further explore these aspects.

## Conclusion

(1) The self-efficacy of college students in exercise can positively predict physical education learning engagement. (2) Exercise self-efficacy can positively predict flow experiences, and flow experiences can positively predict physical education learning engagement. (3) Flow experiences mediate the relationship between exercise self-efficacy and physical education learning engagement.

## Data Availability

The original contributions presented in the study are included in the article/supplementary material, further inquiries can be directed to the corresponding author.
